# Retraction: Predictors and Preventive Strategies of Bleeding After Thyroid Surgery

**DOI:** 10.7759/cureus.r218

**Published:** 2026-01-26

**Authors:** Mohsen Ezzy, Ehab Alameer

**Affiliations:** 1 Department of Surgery, College of Medicine, Jazan University, Jazan, SAU

This article has been retracted by the Editor-in-Chief due to multiple sections of the manuscript closely replicating the wording, structure, and historical narrative of a previously published article (Materazzi G, Ambrosini CE, Fregoli L, et al.: Prevention and management of bleeding in thyroid surgery. Gland Surg. 2017, 6:510-5. 10.21037/gs.2017.06.14). These similarities involve synonym substitution and sentence rearrangement and extend across background discussion, historical quotations, and descriptions of surgical technique. Such overlap exceeds acceptable standards for paraphrasing, particularly given that the apparent source article is cited only once despite being repeatedly relied upon throughout the text.

